# MedAlmighty: enhancing disease diagnosis with large vision model distillation

**DOI:** 10.3389/frai.2025.1527980

**Published:** 2025-08-12

**Authors:** Yajing Ren, Zheng Gu, Wen Liu

**Affiliations:** Artificial Intelligence and Smart Mine Engineering Technology Center, Xinjiang Institute of Engineering, Urumqi, China

**Keywords:** disease diagnosis, large vision model, knowledge distillation, model capacity, domain generalization

## Abstract

**Introduction:**

Accurate disease diagnosis is critical in the medical field, yet it remains a challenging task due to the limited, heterogeneous, and complex nature of medical data. These challenges are particularly pronounced in multimodal tasks requiring the integration of diverse data sources. While lightweight models offer computational efficiency, they often lack the comprehensive understanding necessary for reliable clinical predictions. Conversely, large vision models, trained on extensive general-domain datasets, provide strong generalization but fall short in specialized medical applications due to domain mismatch and limited medical data availability.

**Methods:**

To bridge the gap between general and specialized performance, we propose MedAlmighty, a knowledge distillation-based framework that synergizes the strengths of both large and small models. In this approach, we utilize DINOv2—a pre-trained large vision model—as a frozen teacher, and a lightweight convolutional neural network (CNN) as the trainable student. The student model is trained using both hard labels from the ground truth and soft targets generated by the teacher model. We adopt a hybrid loss function that combines cross-entropy loss (for classification accuracy) and Kullback-Leibler divergence (for distillation), enabling the student model to capture rich semantic features while remaining efficient and domain-aware.

**Results:**

Experimental evaluations reveal that MedAlmighty significantly improves disease diagnosis performance across datasets characterized by sparse and diverse medical data. The proposed model outperforms baselines by effectively integrating the generalizable representations of large models with the specialized knowledge from smaller models. The results confirm improved robustness and accuracy in complex diagnostic scenarios.

**Discussion:**

The MedAlmighty framework demonstrates that incorporating general-domain representations via frozen large vision models—when guided by task-specific distillation strategies—can enhance the performance of lightweight medical models. This approach offers a promising solution to data scarcity and domain gap issues in medical imaging. Future work may explore extending this distillation strategy to other medical modalities and incorporating multimodal alignment for even richer representation learning.

## 1 Introduction

Artificial intelligence (AI) has driven transformation progress in imaging and vision, empowering applications across fields such as autonomous driving, robotics, and healthcare. In the medical domain, computer-aided diagnosis (CAD) has become pivotal for enhancing disease prognosis, enabling early detection, guiding risk stratification, and supporting personalized treatment planning (Edupuganti et al., 2024). However, despite remarkable advances, a persistent bottleneck remains: disparities in medical resource distribution and limited access to high-quality annotated data often hinder the deployment of robust diagnostic systems, especially in under-resourced regions. To address this, medical professionals increasingly rely on diverse imaging modalities—including X-rays, MRI, CT scans, PET, SPECT, and ultrasound—which provide critical insights into patient conditions (Zhu Y. et al., 2024; Arumugam et al., 2024; Miller et al., 2024; Wang X. et al., 2024).

Within the broader landscape of multimodal vision AI (Huo et al., 2025; Yan et al., 2024; Lyu et al., 2024), large-scale models have demonstrated unparalleled generalization capabilities, learning powerful feature representations from vast non-medical datasets (Zheng et al., 2025). Yet, their application to medical imaging remains underexplored, largely due to two key challenges: the scarcity of diverse, high-quality medical data and the complex multimodal nature of diagnostic tasks (Zheng et al., 2022; Chen et al., 2022). While successful classification models often build upon enhanced CNN variants, lightweight models face inherent limitations in capturing sufficient knowledge because of their restricted capacity. Consequently, there has been a shift toward large vision models with deeper architectures and richer parameters, which promise more expressive and generalized features (Zhang W. et al., 2024; Zhu C. et al., 2024; Liao et al., 2025; Zhao et al., 2025; Zhong et al., 2025). However, applying these models directly to medical imaging introduces domain gaps; they are typically pre-trained on natural images, which lack the biological and pathological features essential for accurate medical interpretation.

In the realm of medical image classification, researchers have sought to address data scarcity through three main strategies: image generation and enhancement, transfer learning, and knowledge distillation. For example, GAN-based methods (Feng et al., 2024; MeenaPrakash et al., 2025) synthesize realistic medical images to augment small datasets, improving downstream performance. Pre-training approaches such as RadImageNet (Mei et al., 2022) leverage large radiology datasets to enhance generalizability, while transfer learning techniques (Wang W. et al., 2024) adapt models to specific diagnostic tasks. Knowledge distillation methods (Song et al., 2025) further compress large models into lightweight versions suitable for resource-constrained environments. Despite their individual successes, many of these methods require custom model designs tailored to specific datasets and tasks, incurring significant development costs and limiting scalability.

In the area of transfer learning, Wang W. et al. (2024) propose a DenseNet-based breast cancer classification model that incorporates attention mechanisms and multi-level transfer learning. This model achieves an accuracy of over 84.0%, demonstrating improved efficiency for pathological image analysis. In knowledge distillation, (Song et al. 2025) present a lightweight Shift-MLP-based student model with multi-teacher distillation. Additionally, they introduce a two-stage diagnostic framework that fuses multimodal data and transfers privileged knowledge from teacher to student models, outperforming existing methods in glioma grading and skin lesion classification.

While the aforementioned approaches effectively address the limited sample sizes in medical image datasets, they often rely on custom-designed models tailored to specific datasets and tasks, resulting in high development and deployment costs. In contrast, our goal is to explore ***a more generalizable solution—one that performs consistently across diverse*** ***medical modalities despite data scarcity***. Large vision models, with their substantial parameter capacity and complex architectures, have shown strong generalization and robust feature representation in computer vision, making them promising candidates for this purpose.

Accordingly, we adopt DINOv2 as our base model. However, experiments reveal inconsistent performance across different medical tasks. This may stem from a fundamental domain gap: DINOv2 is pre-trained on natural images, which lack the concept of biological tissue, whereas medical image analysis often relies on distinguishing between normal and abnormal tissues. For example, pneumonia diagnosis in X-rays depends on detecting diffuse pathological changes in lung tissue, while conditions like cardiac tumors or edema are more associated with localized boundary changes—features that align better with edge-sensitive representations learned from natural images. These observations suggest that large vision models alone may not fully capture the nuances of medical data. Therefore, we propose combining the strengths of large vision models with the domain-specific expertise of smaller models to achieve more reliable and adaptable performance.

**Figure 1:**
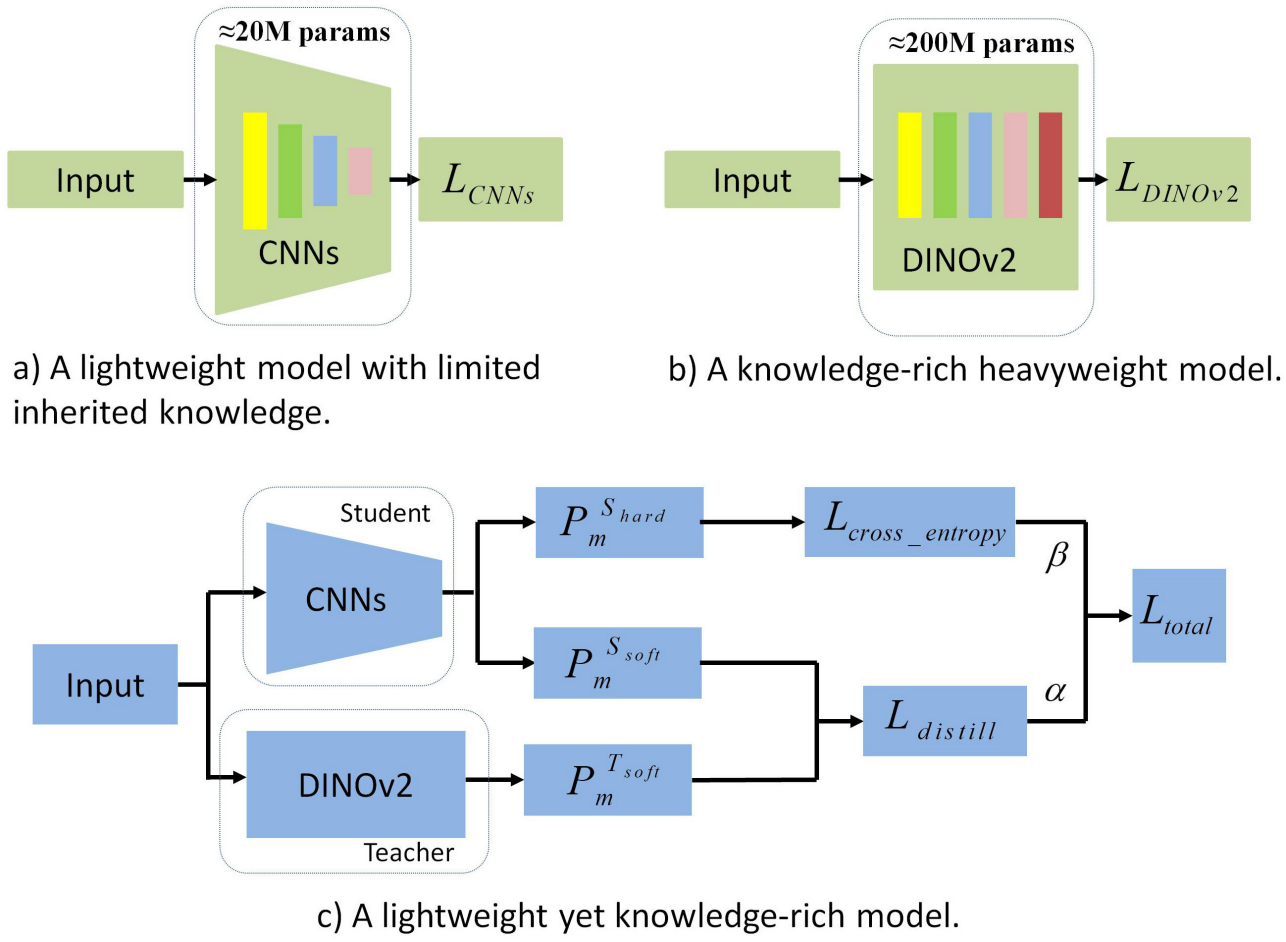
Comparison of generalization and training efficiency between CNNs and DINOv2. This figure provides a comprehensive comparison of CNNs and DINOv2 in terms of generalization and training efficiency. **(a)** Generalization Performance: CNNs struggle with robustness and accuracy on unseen data, while DINOv2 exhibits stronger generalization across diverse tasks due to self-supervised learning. **(b)** Training Efficiency: DINOv2 requires significantly more computational resources and training time, limiting its practicality. **(c)** Synergy Potential: The figure also underscores the advantages of combining CNNs' efficiency with DINOv2's generalization, motivating the integration of both in a unified framework.

To ***bridge the gap between generalizable representation learning and domain-specific*** ***efficiency***, we introduce ***MedAlmighty***, a distillation framework that transfers knowledge from a large vision model (DINOv2) to a compact student model (ResNet), which demonstrated strong performance among baselines on the MedMNISTv2 dataset. As illustrated in Figure 1, the distillation process enables the student model to inherit robust and general features from the teacher while maintaining high classification accuracy on limited medical data. We evaluate MedAlmighty across all 12 modalities in the MedMNISTv2 dataset and compare it against existing lightweight enhancement approaches to validate its effectiveness.

Our key contributions are as follows:

- We present MedAlmighty, a knowledge distillation framework that integrates the robust feature representation of a large vision model (DINOv2) with the lightweight efficiency of a small model (ResNet), enabling effective classification across 12 diverse medical imaging modalities.
- We investigate the use of a frozen DINOv2 backbone with a trainable linear classifier and observe its limitations in domain-specific tasks, motivating the need for knowledge transfer to a smaller, specialized model.
- MedAlmighty leverages the complementary strengths of both large and small models, offering a generalizable and scalable solution for medical image classification under data-scarce and multi-modality conditions.
- We conduct extensive experiments on the MedMNISTv2 dataset and benchmark MedAlmighty against multiple lightweight model enhancement baselines, demonstrating its superior performance and robustness.

## 2 Related work

### 2.1 Supervised learning in medical image analysis

Supervised learning has been widely adopted in medical image analysis, achieving strong performance across various tasks. Recent efforts have focused on enhancing the accuracy and robustness of supervised models, particularly through deep learning approaches. Convolutional neural networks (CNNs) have proven effective in extracting discriminative features from medical images (Liu et al., 2024; Yang et al., 2025; Mishra et al., 2025; Maree et al., 2024; Oyelade et al., 2024). Attention mechanisms have also been incorporated into CNNs to improve classification by emphasizing salient image regions (Takahashi et al., 2024). Supervised learning offers key advantages, including high accuracy with clear labels, the ability to learn complex feature representations from large annotated datasets, and adaptability to diverse clinical tasks such as disease diagnosis and lesion detection. To further boost performance, transfer learning has been widely explored, enabling models to generalize better with limited labeled data. For instance, CNN-based transfer learning approaches have been used to classify benign and malignant breast masses in X-ray images, with enhanced results achieved through model selection, ensemble averaging, and feature concatenation (Han et al., 2024).

More recently, Vision Transformers (ViTs) (Dosovitskiy et al., 2020), originally developed for natural image recognition, have gained attention in medical imaging due to their ability to model long-range dependencies via self-attention. ViT-based models have shown promise in handling complex and heterogeneous data. For example, MedViT (Manzari et al., 2023) integrates local feature extraction with global context modeling and efficient attention mechanisms, demonstrating strong performance on datasets like MedMNIST-2D (Yang et al., 2020). Despite these advances, generalizability remains a significant challenge (Liu et al., 2025; Pacal et al., 2025). Many supervised methods are designed for single-modality inputs and struggle with the multimodal nature of real-world medical data. Moreover, the need for large volumes of expert-labeled data poses practical constraints, as annotation is time-consuming, costly, and may introduce subjectivity. Addressing these limitations is crucial for developing robust, scalable models suited for clinical deployment.

### 2.2 Self-supervised learning in medical image analysis

Self-supervised learning has emerged as a powerful approach in medical image analysis, particularly for addressing the scarcity and cost of annotated data. By leveraging the inherent structure of medical images to generate supervisory signals, it enables the learning of robust feature representations without extensive manual labeling. Many self-supervised methods draw inspiration from Masked Autoencoders (MAE) (He et al., 2021). For instance, ChA-MAEViT (Pham et al., 2025) enhances medical image classification by explicitly modeling cross-channel dependencies via dynamic masking and memory tokens, achieving up to 21.5% higher accuracy than existing MCI-ViTs on microscopy datasets. Similarly,MSMAE (Mao et al., 2025) introduces a supervised attention-driven masking strategy (SAM) to precisely localize and learn lesion-related regions in medical images, achieving SOTA classification accuracy 68.41%–99.60% while reducing FLOPs by 74.08% and inference time by 11.2% compared to MAE.

Autoencoder-based architectures have also been explored, with design improvements addressing class imbalance and enhancing diagnostic accuracy (Xing et al., 2023; Arafa et al., 2023). A lightweight self-supervised learning (SSL) model (Karagoz and Nalbantoglu, 2024) for mammography classification, combining a VAE-based pretext task for feature learning and a 3-layer CNN downstream network, achieving 0.94–0.99 AUC with only 228 parameters and 204.95K FLOPs on INbreast/MIAS datasets. A notable example, ViT-AE++, introduces novel loss functions for self-reconstruction and contrastive learning to enhance representation quality (Prabhakar et al., 2023). More broadly, contrastive learning has proven effective in distinguishing disease features from normal structures without labeled data (Zhang X. et al., 2024; Jiao et al., 2024; Kumar and Marttinen, 2024; Li M. et al., 2025; Li Q. et al., 2025; Xu and Wong, 2025; Liu et al., 2022; Nguyen et al., 2024). Despite its advantages, self-supervised learning faces limitations. Without task-specific labels, models may miss subtle features essential for accurate diagnosis. Moreover, many approaches are tailored to specific diseases or modalities, often requiring significant computation and long training times. Their adaptability to heterogeneous datasets—common in medical imaging due to varying acquisition protocols—also remains limited.

### 2.3 Large vision models

Large vision models have advanced significantly in computer vision, drawing inspiration from architectures such as CLIP (Radford et al., 2021), MAE, ViT, and Beit (Bao et al., 2021; Peng et al., 2022). Most adopt the Transformer architecture—particularly Vision Transformer (ViT)—and are trained using diverse paradigms, including supervised learning [e.g., DeiT-III (Touvron et al., 2022)], text-image contrastive learning (e.g., OpenCLIP), and self-supervised learning [e.g., DINO (Caron et al., 2021), DINOv2 (Oquab et al., 2023)]. Numerous variants have been proposed to improve performance and efficiency. For example, Stream-ViT (Pan et al., 2025) dynamically integrates streamlined high-to-low resolution convolutions with self-attention, enhancing model capacity and efficiency . These models have set benchmarks across vision tasks and motivated the scaling of model size and data volume. Following their success on natural image datasets like ImageNet, interest is growing in adapting these models to medical imaging. Researchers are exploring whether representations learned during large-scale pre-training can benefit medical tasks such as classification, segmentation, and diagnosis—especially where labeled data is limited (Huix et al., 2024). Early results suggest that zero-shot and few-shot capabilities of vision-language models (e.g., CLIP variants) show promise for lesion recognition and medical report generation, enabling more flexible and scalable diagnostic tools. Emerging frameworks support alignment between medical tasks and large models. Domain-specific pre-training on datasets such as MIMIC-CXR and MedMNIST has been explored to better adapt general models to medical data. Additionally, methods like lightweight adapters, prompt tuning, and hybrid architectures aim to integrate pre-trained models into clinical pipelines with reduced computational overhead. Despite these advances, challenges remain. Medical images differ markedly from natural images in modality, appearance, and annotation granularity. Moreover, clinical deployment demands greater interpretability, robustness, and domain adaptation. Addressing these issues is essential to fully realize the potential of large vision models in medical image analysis.

## 3 Methods

### 3.1 Problem definition

To address the challenges of medical image classification under data-scarce conditions, we propose a knowledge distillation framework that integrates the robust, generalizable feature extraction capabilities of large-scale vision models with the fine-tuned, domain-specific expertise of smaller models trained on medical datasets. Formally, we define the medical dataset as $D={\left\{\left(X_{i},y_{i}\right)\right\}}_{i=1}^{n}$, where *n* is the number of labeled samples, $X_{i}\in {\mathbb{R}}^{C_{m}\times H\times W}$ represents a 2D medical image with *C*_*m*_ channels and spatial dimensions *H*×*W*, and *y*_*i*_ is the corresponding ground-truth label.

In this framework, the teacher model *T* is a large, pre-trained vision network (e.g., DINOv2) with a vast parameter space, capable of learning generalizable visual features. The student model *S* is a compact, lightweight network (e.g., CNN-based) specifically adapted to medical data. The distillation process aims to guide the student model *S* to accurately predict labels ŷ_*i*_ by learning from both the ground-truth labels and the rich feature representations $F^{T}$ provided by the teacher model. By bridging the gap between general visual knowledge and domain-specific patterns, the proposed approach enables the student model to combine broad, transferable features with detailed, task-relevant refinements, ultimately improving efficiency and accuracy in medical image classification tasks.

### 3.2 Architecture

To leverage the advantages of large vision models while incorporating domain-specific knowledge from smaller medical datasets, we have designed a distillation framework that combines the powerful feature extraction capabilities of large models with the fine-grained domain-specific features learned by smaller models.

For an input sample *X*_*i*_, it is simultaneously passed through both the teacher model *T*_*DINOv*2_ and the student model *S*_*CNNs*_, generating output features $F_{1}^{T_{DINOv2}}$ and $F_{1}^{S_{CNNs}}$. These output features are then processed through a linear layer *f*_*Linear*_(·) to obtain the output vectors:


$$\left\{ \begin{array}{l} Z_{1}^{T_{DINOv2}}=\left\{z_{11}^{T_{DINOv2}},z_{12}^{T_{DINOv2}},...,z_{1m}^{T_{DINOv2}}\right\}\\ \;Z_{1}^{S_{CNNs}}=\left\{z_{11}^{S_{CNNs}},z_{12}^{S_{CNNs}},...,z_{1m}^{S_{CNNs}}\right\} \end{array}\right.$$

The dimensions of $Z_{1}^{T_{DINOv2}}$ and $Z_{1}^{S_{CNNs}}$ are both *m*, where *m* represents the number of classes. We then apply the softmax function *f*_*softmax*_(·) to convert the output vectors $Z_{1}^{T_{DINOv2}}$ and $Z_{1}^{S_{CNNs}}$ into probability distribution vectors $P_{m}^{T_{soft}}$ and $P_{m}^{S_{soft}}$, respectively. Each element $P_{m}^{soft}$ represents the probability that the input belongs to class *m*:


$$P_{m}^{soft}=\frac{e^{z_{m}/t}}{\sum_{j=1}^{m}e^{z_{j}/t}},$$

where *t* is the distillation temperature, controlling the softness of the label distribution between the teacher and student networks. A higher temperature value *t* smooths the soft labels from the teacher model, providing more informative learning signals to the student model, which helps improve its generalization ability. By increasing *t*, the student model can more easily learn from the teacher's knowledge, leading to improved performance.

To enhance the generalization ability of the CNN student model in medical image classification, we minimize the Kullback-Leibler (KL) divergence between the soft labels $P_{m}^{soft}$ of the student and teacher models. KL divergence measures the difference between two probability distributions and is defined as:


$$D_{KL}\left(P_{m}^{S_{soft}}||P_{m}^{T_{soft}}\right)=-t^{2}\overset{m}{\underset{i=1}{\sum }}P^{S_{soft}}\left(i\right)\log\frac{P^{S_{soft}}\left(i\right)}{P^{T_{soft}}\left(i\right)}.$$

Here, *t* represents the distillation temperature. By minimizing this KL divergence, the student model optimizes its prediction while acquiring richer knowledge from the teacher model, thereby improving both performance and generalization. Thus, the distillation loss *L*_distill_ is introduced to minimize the KL divergence and enable the CNN model to better emulate the knowledge distribution of the teacher model *T*_*DINOv*2_ for medical image classification. The distillation loss is represented as:


$$L_{\text{distill}}=D_{KL}\left(P^{T_{soft}},P^{S_{soft}}\right).$$

To effectively combine the strengths of both the teacher and student models, the same training dataset is used. The student model *S*_*CNNs*_ is trained on the true labels of the dataset ${\left\{y_{i}\right\}}_{i=1}^{n}$. For each input sample *X*_*i*_, the student model generates the output feature $F_{2}^{S_{CNNs}}$, which is then passed through a linear layer *f*_*Linear*_(·) to produce the vector:


$$Z_{2}^{S_{CNNs}}=\left\{z_{21}^{S_{CNNs}},z_{22}^{S_{CNNs}},...,z_{2m}^{S_{CNNs}}\right\}$$

where *m* is the number of classes. The predicted probability distribution of the student network, with the distillation temperature *t* = 1, is given by:


$$P_{m}^{S_{hard}}=\frac{e^{z_{m}}}{\sum_{j=1}^{m}e^{z_{j}}},$$

where each element $P_{m}^{S_{hard}}$ represents the probability of the input belonging to class *m*. The student network minimizes the cross-entropy loss between the predicted probabilities and the true labels *y*_*i*_ in the medical dataset ${\left(X_{i},y_{i}\right)}_{i=1}^{n}$. The cross-entropy loss is computed as:


$$L_{\text{cross\_entropy}}=-\underset{i}{\sum }y_{i}\log\left(P_{m}^{S_{hard}}\right),$$

where *y*_*i*_ is the true label of the *i*-th sample.

To integrate knowledge from the teacher network and align with the student network's training objective, we define the total loss *L*_total_ as a weighted sum of the cross-entropy loss *L*_cross_entropy_ and the distillation loss *L*_distill_. This total loss is expressed as:


$$L_{\text{total}}=\alpha \cdot L_{\text{cross\_entropy}}+\beta \cdot L_{\text{distill}},$$

where α and β are hyperparameters that control the relative importance of the two loss functions, with β = 1−α. Specifically, α controls the weight of the cross-entropy loss, while β determines the weight of the distillation loss. Adjusting α and β allows for balancing the incorporation of teacher network knowledge and the maintenance of classification accuracy.

When α is larger, the student model focuses more on matching the true labels, thereby improving classification accuracy. Conversely, increasing β emphasizes the distillation loss, enabling the student model to learn more effectively from the teacher network's knowledge. This facilitates the transfer of prior knowledge from the teacher, enhancing the generalization ability of the student model. By carefully tuning α and β, an optimal balance can be achieved that maximizes the use of the teacher network's knowledge while maintaining strong classification performance. The specific values of these hyperparameters should be determined through experimentation and tuning based on the task and dataset.

## 4 Experiments

### 4.1 Dataset

MedMNIST v2 is a large-scale collection of standardized biomedical images, designed similarly to the widely used MNIST dataset. It includes 12 different datasets for 2D images, with detailed descriptions provided in Table 1. All images in MedMNIST v2 have been pre-processed to a uniform size of 28 × 28 pixels (2D). The dataset spans a wide range of primary data modalities in biomedical imaging and is specifically designed for lightweight classification tasks on 2D images. MedMNIST v2 accommodates datasets of varying scales, ranging from as few as 100 samples to as many as 100,000 samples. It supports various classification tasks, including binary/multi-class, ordinal regression, and multi-label classification. In total, MedMNIST v2 contains an impressive 708,069 2D images, providing a rich and diverse set of data for experimental analysis.

**Table 1:** Detailed information of the medical image dataset.

**MedMNIST2D**	**PathMNIST**	**ChestMNIST**	**DermaMNIST**	**OCTMNIST**	**PneumoniaMNIST**	**RetinaMNIST**
Data Modality	Colon Pathology	Chest X-Ray	Dermatoscope	Retinal OCT	Chest X-Ray	Fundus Camera
**Tasks(Labels)**	**Multi-class (9)**	**Multi-label (14) Binary-class (2)**	**Multi-class (7)**	**Multi-class (4)**	**Binary-class (2)**	**Ordinal regression (5)**
Samples	107,180	112,120	10,015	109,309	5,856	1,600
Training	89,996	78,468	7,007	97,477	4,708	1,080
Validation	10,004	11,219	1,003	10,832	524	120
Test	7,180	22,433	2,005	1,000	624	400
**MedMNIST2D**	**BreastMNIST**	**BloodMNIST**	**TissueMNIST**	**OrganAMNIST**	**OrganCMNIST**	**OrganSMNIST**
Data modality	Breastultrasound	Blood cellmicroscope	Kidney cortex Microscope	Abdominal CT	Abdominal CT	Abdominal CT
**Tasks (Labels)**	**Binary-class (2)**	**Multi-class (8)**	**Multi-class (8)**	**Multi-class (11)**	**Multi-class (11)**	**Multi-class (11)**
Samples	780	17,092	236,386	58,830	23,583	25,211
Training	546	11,959	165,466	34,561	12,975	13,932
Validation	78	1,712	23,640	6,491	2,392	2,452
Test	156	3,421	47,280	17,778	8,216	8,827

The table presents various medical image modalities, including 2D medical images, pathology, chest X-ray, dermoscopy, retinal OCT, pneumonia X-ray, and fundus camera. Each modality has different tasks and labels associated with it. The table also provides the sample count for each dataset, as well as the division into training, validation, and test sets.

### 4.2 Implementation details

#### 4.2.1 Training process

All experiments are conducted using NVIDIA RTX 3090 GPUs within a PyTorch framework. Input images are uniformly resized to 224 × 224 pixels, and the number of input channels is fixed at 3. For grayscale images, we replicate the single channel to form RGB inputs. To identify the best-performing models on the MedMNIST v2 dataset, we adopt an early stopping strategy based on validation performance. We evaluate three convolutional neural networks: ResNet50, SENet50, and SKNet50. During training, we employ a multi-step learning rate schedule, starting with an initial learning rate of 0.001. The learning rate is reduced by a factor of 0.1 at epochs 50 and 75. The models are optimized using the Adam optimizer with a batch size of 128 and trained for 100 epochs. For multi-label and binary classification tasks, we use the binary cross-entropy loss function; for all other tasks, standard cross-entropy loss is applied.

For experiments involving the DINOv2 framework, we assess the performance of three backbone variants: DINOv2-ViT-S/14, DINOv2-ViT-B/14, and DINOv2-ViT-L/14, across all 12 datasets in MedMNIST v2. In these experiments, the DINOv2 backbone remains frozen, and only the classification head is fine-tuned. No data augmentation is applied. The models are trained using the Adam optimizer with an initial learning rate of 0.001 and a batch size of 32. The learning rate schedule mirrors that of the CNN models, with reductions at epochs 50 and 75. Cross-entropy loss is used for all classification tasks. For the MedAlmighty experiments, we adopt the same training settings as used in the DINOv2 experiments. Specifically, the best-performing DINOv2 model with a ViT-B/14 backbone is selected as the teacher, and ResNet50 is used as the student model. We explore the impact of knowledge distillation on classification performance across the MedMNIST v2 datasets.

#### 4.2.2 Evaluation metrics

To enable a fair comparison with the baseline methods used in MedMNIST v2, we adopt the same evaluation metrics: Area Under the Curve (AUC) and Accuracy (ACC).

**AUC** serves as a comprehensive performance metric that captures the trade-off between the true positive rate and false positive rate across various threshold settings. It is particularly useful for evaluating classification models on imbalanced datasets, where traditional accuracy metrics may be misleading. A higher AUC indicates stronger discriminatory ability.

**ACC** measures the proportion of correctly classified samples across the entire dataset. As a straightforward and intuitive metric, it provides a general assessment of a model's overall classification accuracy. Higher ACC values denote better performance.

### 4.3 Experimental results

#### 4.3.1 Inconsistent improvement of DINOv2 compared to CNNs

We evaluate three CNN-based architectures—ResNet50, SENet50, and SKNet50—and compare them against transformer-based models from the DINOv2 family: DINOv2-ViT-S/14, DINOv2-ViT-B/14, and DINOv2-ViT-L/14, as well as our proposed MedAlmighty framework. Their performances in terms of AUC and ACC across the 12 MedMNIST v2 datasets are summarized in Table 2.

**Table 2:** Performance comparison of CNN, DINOv2 and CA-MKD models on 12 MedMNIST v2 datasets.

**Methods**	**PathMNIST**		**ChestMNIST**		**DermaMNIST**		**OCTMNIST**	
	**AUC**	**ACC**	**AUC**	**ACC**	**AUC**	**ACC**	**AUC**	**ACC**
ResNet-50	0.978 ± 0.007	0.854 ± 0.028	0.768 ± 0.004	0.947 ± 0.001	0.901 ± 0.011	0.721 ± 0.011	0.948 ± 0.016	0.776 ± 0.002
SENet-50	0.982 ± 0.002	0.863 ± 0.015	**0.774** **±0.01**	0.948 ± 0.003	0.989 ± 0.004	0.731 ± 0.007	0.949 ± 0.014	0.780 ± 0.058
SKNet-50	**0.986** **±0.004**	0.844 ± 0.014	0.768 ± 0.002	0.947 ± 0.001	0.986 ± 0.003	0.719 ± 0.015	0.942 ± 0.017	0.770 ± 0.006
DINOV2-vits14	0.986 ± 0.005	0.857 ± 0.009	0.666 ± 0.016	0.946 ± 0.007	0.903 ± 0.021	0.727 ± 0.037	0.924 ± 0.010	0.659 ± 0.026
DINOV2-vitb14	0.981 ± 0.005	0.870 ± 0.051	0.654 ± 0.015	0.943 ± 0.005	0.905 ± 0.025	0.725 ± 0.034	0.929 ± 0.015	0.629 ± 0.017
DINOV2-vitl14	0.979 ± 0.002	0.862 ± 0.032	0.649 ± 0.022	**0.962** **±0.007**	0.901 ± 0.029	0.732 ± 0.042	0.933 ± 0.011	0.663 ± 0.021
CA-MKD	0.966 ± 0.002	0.832 ± 0.022	0.762 ± 0.004	0.947 ± 0.001	0.898 ± 0.001	0.700 ± 0.008	0.933 ± 0.01	0.766 ± 0.002
MedAlmighty	0.955 ± 0.03	**0.871** **±0.02**	0.643 ± 0.05	0.927 ± 0.02	**0.905** **±0.007**	**0.735** **±0.005**	**0.951** **±0.008**	**0.781** **±0.003**
**Methods**	**BreastMNIST**		**BloodMNIST**		**TissueMNIST**		**PneumoniaMNIST**	
	**AUC**	**ACC**	**AUC**	**ACC**	**AUC**	**ACC**	**AUC**	**ACC**
ResNet-50	0.883 ± 0.015	**0.843** **±0.004**	0.994 ± 0.001	0.950 ± 0.012	0.931 ± 0.005	0.680 ± 0.011	0.962 ± 0.005	0.884 ± 0.007
SENet-50	0.876 ± 0.001	0.827 ± 0.006	**0.995** **±0.002**	0.960 ± 0.005	0.926 ± 0.004	0.687 ± 0.013	0.949 ± 0.016	0.816 ± 0.058
SKNet-50	0.859 ± 0.031	0.818 ± 0.009	0.993 ± 0.002	0.956 ± 0.014	0.925 ± 0.003	0.687 ± 0.005	0.958 ± 0.006	0.839 ± 0.018
DINOV2-vits14	0.871 ± 0.015	0.853 ± 0.012	0.992 ± 0.004	0.926 ± 0.016	0.902 ± 0.007	0.610 ± 0.015	0.963 ± 0.02	0.893 ± 0.016
DINOV2-vitb14	**0.893** **±0.024**	0.827 ± 0.008	0.993 ± 0.006	0.930 ± 0.007	0.916 ± 0.004	0.647 ± 0.018	0.962 ± 0.017	0.890 ± 0.021
DINOV2-vitl14	0.853 ± 0.017	0.842 ± 0.022	0.994 ± 0.009	0.928 ± 0.010	0.911 ± 0.002	0.632 ± 0.021	**0.963** **±0.009**	0.872 ± 0.017
CA-MKD	0.877 ± 0.01	0.830 ± 0.002	0.991 ± 0.001	0.949 ± 0.011	0.928 ± 0.002	0.662 ± 0.001	0.962 ± 0.001	0.882 ± 0.003
MedAlmighty	0.837 ± 0.03	0.833 ± 0.01	**0.995** **±0.002**	**0.960** **±0.004**	**0.932** **±0.002**	**0.693** **±0.005**	0.946 ± 0.003	**0.915** **±0.003**
**Methods**	**RetinaMNIST**		**OrganAMNIST**		**OrganCMNIST**		**OrganSMNIST**	
	**AUC**	**ACC**	**AUC**	**ACC**	**AUC**	**ACC**	**AUC**	**ACC**
ResNet-50	0.713 ± 0.008	0.500 ± 0.010	0.997 ± 0.001	0.944 ± 0.002	0.991 ± 0.001	0.910 ± 0.001	0.963 ± 0.003	0.766 ± 0.017
SENet-50	0.729 ± 0.014	**0.524** **±0.018**	0.997 ± 0.001	0.941 ± 0.003	0.992 ± 0.001	0.905 ± 0.002	0.963 ± 0.001	0.765 ± 0.019
SKNet-50	0.728 ± 0.007	0.520 ± 0.006	0.997 ± 0.002	0.935 ± 0.005	0.991 ± 0.002	0.907 ± 0.001	0.954 ± 0.032	0.778 ± 0.006
DINOV2-vits14	**0.738** **±0.022**	0.514 ± 0.020	0.992 ± 0.005	0.880 ± 0.008	0.982 ± 0.010	0.863 ± 0.025	0.961 ± 0.031	0.754 ± 0.006
DINOV2-vitb14	0.735 ± 0.038	0.489 ± 0.020	0.989 ± 0.001	0.889 ± 0.002	0.981 ± 0.005	0.851 ± 0.001	0.962 ± 0.029	0.750 ± 0.012
DINOV2-vitl14	0.730 ± 0.047	0.450 ± 0.025	0.991 ± 0.002	0.892 ± 0.002	0.978 ± 0.007	0.842 ± 0.001	0.960 ± 0.017	0.748 ± 0.011
CA-MKD	0.701 ± 0.003	0.489 ± 0.006	0.997 ± 0.004	0.931 ± 0.002	0.991 ± 0.001	0.908 ± 0.001	0.954 ± 0.001	0.744 ± 0.011
MedAlmighty	0.645 ± 0.1	0.510 ± 0.025	**0.998** **±0.001**	**0.952** **±0.002**	**0.994** **±0.001**	**0.915** **±0.002**	**0.975** **±0.001**	**0.782** **±0.001**

Values represent mean ± standard deviation over 3 independent runs (highest metric per dataset in bold).

The results reveal that both CNN and DINOv2 models exhibit varying performance across different medical image classification tasks. Among the CNN models, ResNet50 consistently outperforms SENet50 and SKNet50 on most datasets. However, DINOv2 only shows marginal improvements on a subset of datasets—namely, DermaMNIST, RetinaMNIST, and PathMNIST. Although DINOv2 achieves competitive results in some cases, its overall performance is inconsistent and does not consistently surpass that of the CNN models.

**Figure 2:**
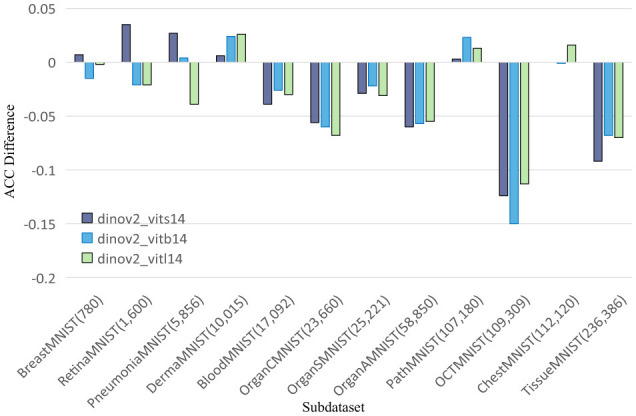
Comparing AUC values of DINOv2-ViTs14 with ResNet18, DINOv2-ViTb14 with ResNet50, and DINOv2-ViTl14 with ResNet50 on 12 MedMNIST datasets. Results are based on experiments using MedMNISTV2, where all models were evaluated on 224 × 224 images.

**Figure 3:**
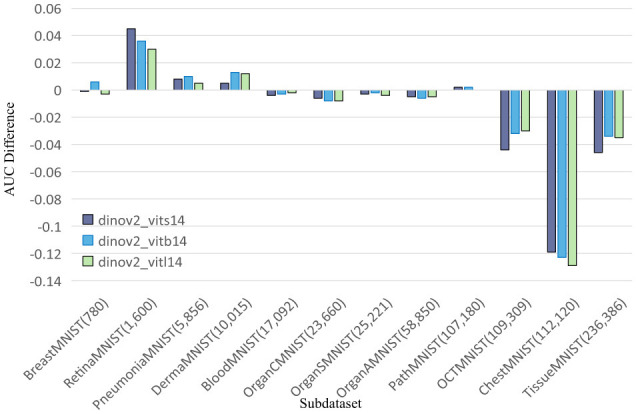
Comparing ACC values of DINOv2-ViTs14 with ResNet18, DINOv2-ViTb14 with ResNet50, and DINOv2-ViTl14 with ResNet50 on 12 MedMNIST datasets. Results are based on experiments using MedMNISTV2, where all models were evaluated on 224 × 224 images.

To further explore this, we compare DINOv2-ViT-B/14 directly with ResNet50 using AUC and ACC metrics, as shown in Figures 2, 3, respectively. These comparisons indicate that DINOv2-ViT-B/14 offers improvements in only a limited number of datasets, and in several cases, its performance falls short of that achieved by ResNet50.

In summary, our findings demonstrate that while DINOv2 can provide benefits on certain datasets, it fails to deliver consistent improvements over CNN models. ResNet50 remains a strong baseline, outperforming both SENet50 and SKNet50, and often matching or exceeding the performance of DINOv2 models in terms of both AUC and ACC across the MedMNIST v2 classification tasks.

#### 4.3.2 CA-MKD vs. MedAlmighty comparison

Additionally, we benchmark against CA-MKD (Zhang et al., 2022) (Confidence-Aware Multi-Teacher Knowledge Distillation), which employs three ResNet32x4 teachers (138M total params) to distill knowledge into a lightweight MobileNetV2 (3.4M params). Their performances in terms of AUC and ACC across the 12 MedMNIST v2 datasets are summarized in Table 2. While multi-teacher distillation incorporates features from multiple teacher models, it merely expands feature quantity without ensuring comprehensive coverage. In contrast, our MedAlmighty method incorporates more comprehensive feature considerations during classification. Experimental results on this dataset demonstrate unsatisfactory performance of the selected multi-teacher distillation model—likely attributable to the identical ResNet32x4 architecture shared by all three teacher models, which constrained its effectiveness. This raises the essential consideration of model selection when applying multi-teacher distillation to target datasets for classification tasks. Furthermore, multi-teacher distillation imposes significantly higher computational demands during training. Compared to this approach, our method demonstrates superior performance and efficiency.

#### 4.3.3 Enhancing classification performance with MedAlmighty

To investigate how to improve the classification performance of CNNs and DINOv2 in medical imaging, we adopt a knowledge distillation approach by transferring learned representations from DINOv2 to ResNet50. This results in our proposed framework, *MedAlmighty*, whose performance across the MedMNIST v2 datasets is presented in Table 2. Experimental results demonstrate that MedAlmighty consistently outperforms both standalone CNNs and DINOv2 on the majority of datasets. This performance gain can be attributed to the integration of DINOv2's rich visual representation capabilities and the efficiency of CNNs, which typically have lower parameter counts than large-scale vision models. The parameter counts for CNN models, DINOv2, and MedAlmighty are summarized in Table 3. These findings highlight the effectiveness of leveraging knowledge distillation from large vision models to enhance lightweight CNNs for medical image classification. Notably, MedAlmighty achieves these improvements without the use of additional data augmentation techniques, which are often computationally intensive. This demonstrates that our distillation-based approach can significantly improve classification performance while maintaining training efficiency. Overall, MedAlmighty offers a promising direction for applying large vision models like DINOv2 in the medical domain, particularly in scenarios with limited labeled data. By efficiently transferring generalizable knowledge, it enhances classification accuracy while preserving the compactness and interpretability of CNN architectures.

**Table 3:** Model parameters are measured in millions.

**Model**	**ResNet50**	**SENet50**	**SKNet50**	**DINOV2-ViTs14**	**DINOV2-ViTb14**	**DINOV2-ViTl14**	**MedAlmighty**
Model parameter count	23M	26M	23M	22M	86M	304M	23M

#### 4.3.4 Parameter ablation experiments

**Figure 4:**
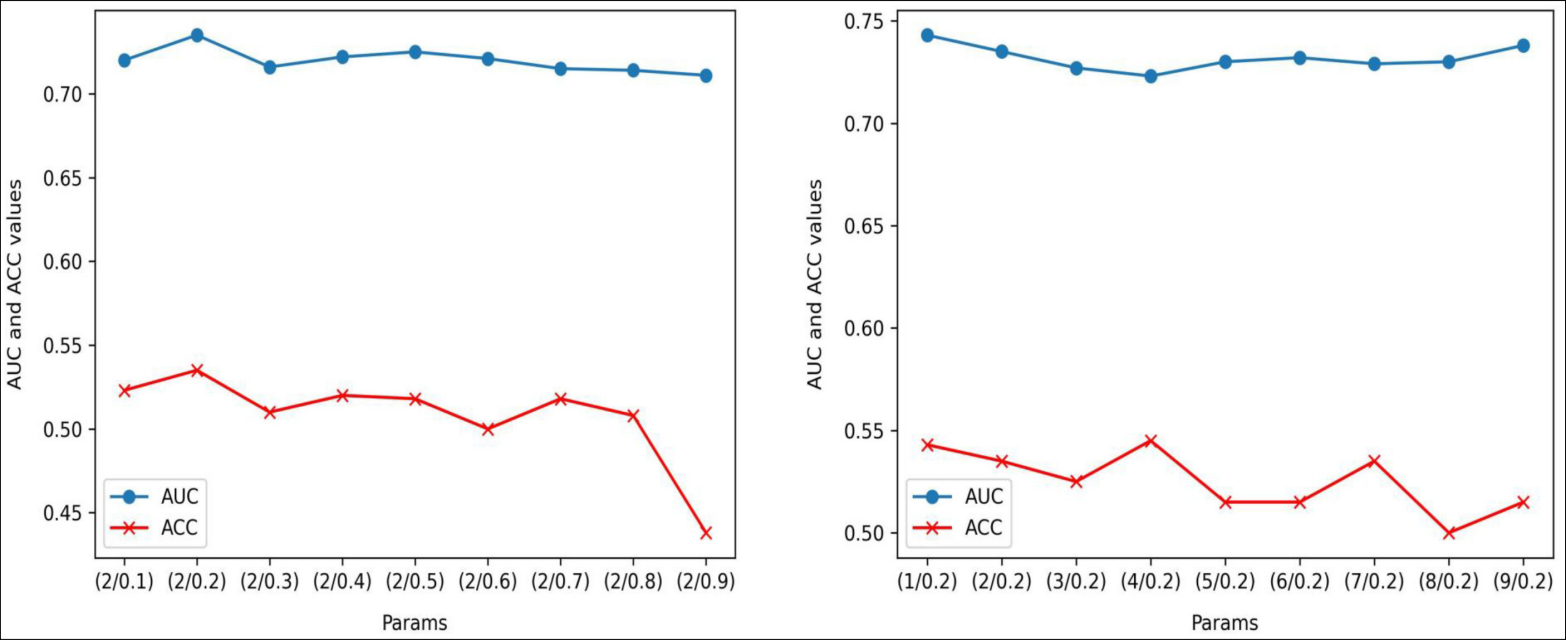
Performance evaluation on RetinaMNIST: AUC and ACC performance of ( *t*/α) with *t* = 2; AUC and ACC performance of (*t*/α) with α=0.2.

In the parameter ablation experiments for MedAlmighty, we explored the effects of varying the distillation temperature *t* and the hyperparameter α across three different combinations. In the first group, we set the distillation temperature *t* = 2.0 and α = 0.2. In the second group, *t* = 5.0 and α = 0.5, and in the third group, *t* = 8.0 and α = 0.8. The results, as shown in Table 4, revealed that the model performed best when both the distillation temperature *t* and α were set to lower values. In further experiments, we fixed the distillation temperature *t* at 2.0 and varied α from 0.1 to 0.9 and we then fixed α at 0.2 and varied *t* from 1 to 9, as shown in Figure 4. The analysis of these experiments indicated that MedAlmighty exhibited superior performance when the distillation temperature *t* was set to a lower value and α was within the range of 0 to 0.5. Furthermore, varying the distillation temperature had little impact on the results when α was kept at a smaller value.

**Table 4:** AUC and ACC results of distilling ResNet50 with DINOv2 ViTb14 on 12 MedMNIST datasets, with varying distillation temperature *t* and α parameters( *t*/α).

**Methods**	**PathMNIST**		**ChestMNIST**		**DermaMNIST**		**OCTMNIST**		**PneumoniaMNIST**		**RetinaMNIST**	
	**AUC**	**ACC**	**AUC**	**ACC**	**AUC**	**ACC**	**AUC**	**ACC**	**AUC**	**ACC**	**AUC**	**ACC**
MedAlmighty (2.0/0.2)	**0.975**	0.883	**0.686**	0.946	**0.911**	0.735	0.954	0.781	**0.946**	**0.915**	**0.735**	**0.535**
MedAlmighty (5.0/0.5)	0.968	0.878	0.671	**0.947**	0.910	**0.738**	**0.965**	**0.804**	0.917	0.909	0.723	0.515
MedAlmighty (8.0/0.8)	0.962	**0.913**	0.642	**0.947**	0.894	0.726	0.946	0.783	0.875	0.638	0.718	0.458
**Methods**	**BreastMNIST**		**BloodMNIST**		**TissueMNIST**		**OrganAMNIST**		**OrganCMNIST**		**OrganSMNIST**	
	**AUC**	**ACC**	**AUC**	**ACC**	**AUC**	**ACC**	**AUC**	**ACC**	**AUC**	**ACC**	**AUC**	**ACC**
MedAlmighty (2.0/0.2)	**0.857**	**0.833**	0.995	**0.962**	**0.932**	**0.693**	**0.998**	**0.952**	**0.994**	0.915	**0.975**	0.783
MedAlmighty (5.0/0.5)	0.834	0.814	**0.997**	0.953	0.931	0.690	0.996	0.926	0.989	**0.921**	**0.975**	**0.789**
MedAlmighty (8.0/0.8)	0.699	0.513	0.994	0.945	0.925	0.683	0.996	0.925	0.988	0.902	0.971	0.782

Bold values represent the better-performing AUC/ACC results among three parameter configurations within the same data subset.

#### 4.3.5 Qualitative visualization

**Figure 5:**
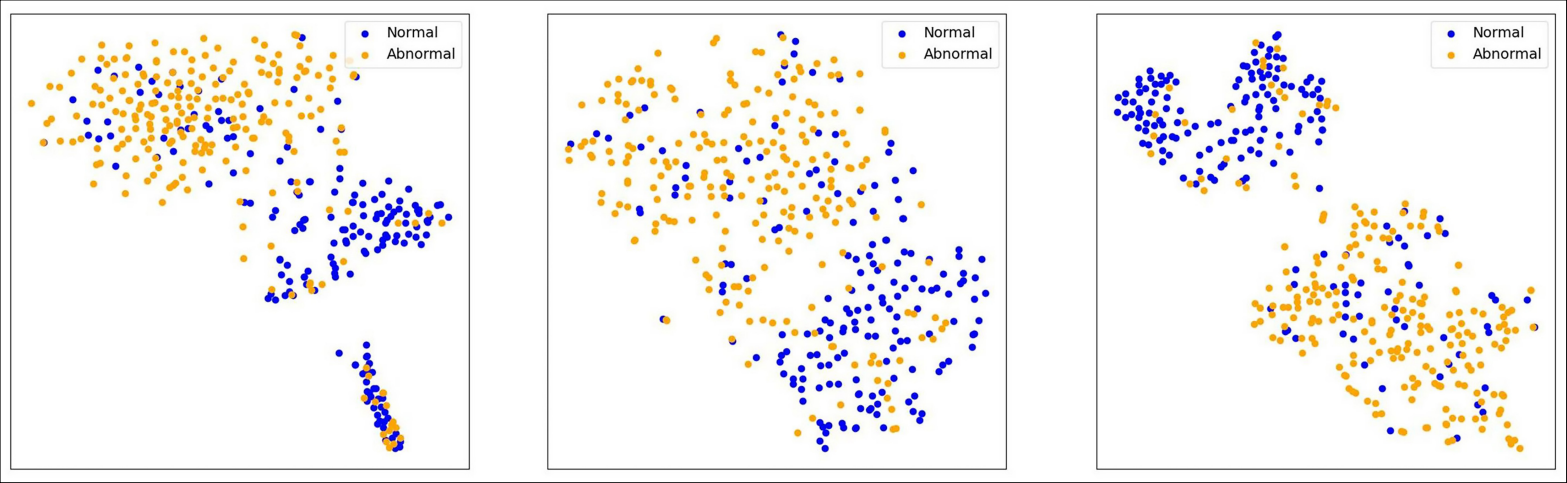
t-SNE visualization of features (ResNet50, DINOV2-ViTb14, MedAlmighty).

To compare the performance differences among three models—ResNet50, DINOv2-ViT-B/14, and MedAlmighty—we visualize the t-SNE plots of normal and abnormal samples from the RetinaMNIST dataset. The results are presented in Figure 5. For normal samples, ResNet50 demonstrates a better clustering effect, with similar samples grouped closely together, forming tight clusters. DINOv2-ViT-B/14 also exhibits some degree of clustering, but there is noticeable separation between different regions of normal samples. In contrast, MedAlmighty shows the most pronounced clustering effect, with clear separation and well-defined clusters of normal samples. For abnormal samples, ResNet50 displays slightly weaker clustering. Some abnormal samples are grouped with normal samples, although many remain distinguishable. DINOv2-ViT-B/14 shows more separation between normal and abnormal samples, but the clustering effect is less distinct. MedAlmighty, however, excels in distinguishing abnormal samples from normal ones, with abnormal samples forming a separate, clearly defined region, distinct from the normal samples. Through qualitative analysis, it is evident that MedAlmighty outperforms both ResNet50 and DINOv2-ViT-B/14 in terms of t-SNE visualization of normal and abnormal samples on the RetinaMNIST dataset. It effectively separates normal and abnormal samples, demonstrating its higher potential and application value for medical image classification tasks.

**Figure 6:**
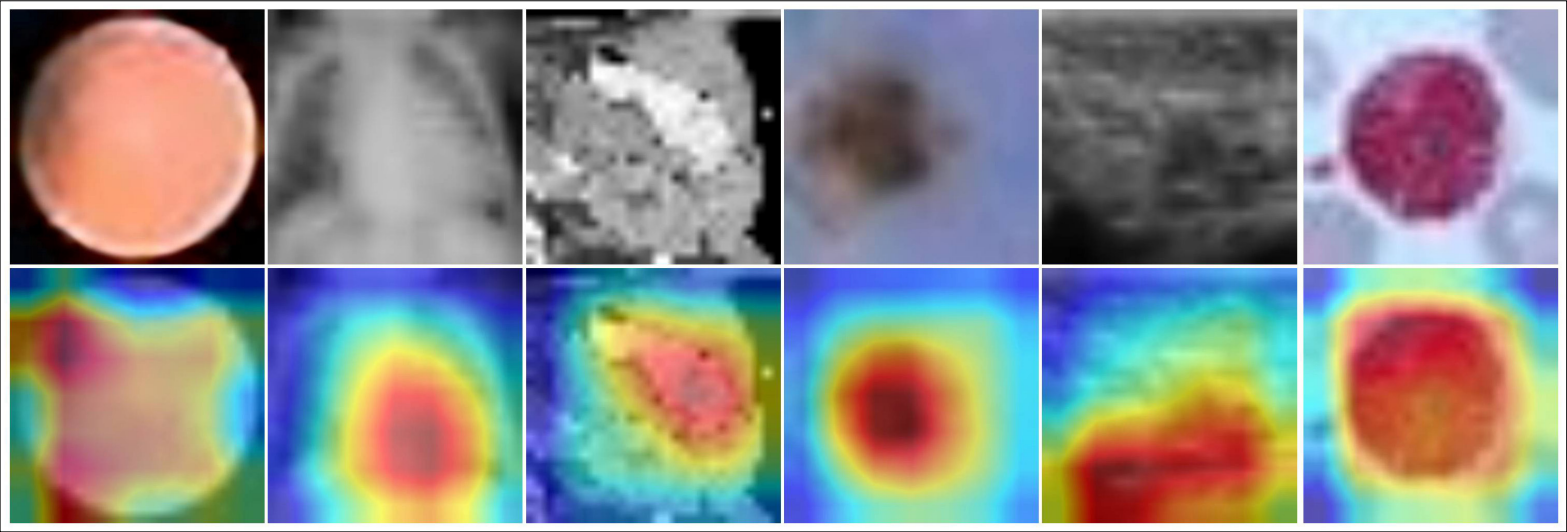
Input images **(top)** and heatmaps **(bottom)**. Color intensity reflects the relative importance of image regions for the model's classification.

Figure 6 showcases heatmap visualizations from a deep learning-based image classification task, demonstrating the model's robust ability to focus on key image features. The top row presents the original input images across different data categories, while the bottom row shows the corresponding heatmaps. The color-coded heatmaps (with red indicating high attention and blue indicating low attention) clearly highlight the areas the model finds most significant for classification. This ability to effectively identify and focus on critical regions underscores the model's strong interpretability and proficiency in understanding complex image patterns.

## 5 Conclusion

The scarcity of diverse, well-annotated medical data and the inherent complexity of disease diagnosis across various imaging modalities underscore the need for innovative solutions in medical image analysis. In this work, we proposed *MedAlmighty*, a framework that integrates the robust generalization capabilities of large pre-trained vision models with the domain-specific knowledge captured by lightweight CNNs, through the technique of knowledge distillation. By distilling knowledge from DINOv2 into ResNet50, MedAlmighty successfully combines the broad semantic understanding of large vision models with the efficiency and specialization of smaller models. This approach addresses the capacity limitations of CNNs while avoiding the computational burden of fine-tuning large models end-to-end. Although initial results from frozen large models with only a trainable linear head showed limited performance, our method effectively bridges this performance gap. Extensive experiments on the MedMNIST v2 benchmark demonstrate that MedAlmighty consistently outperforms both standalone CNNs and large models across a wide range of medical classification tasks. Notably, it achieves these improvements without relying on computationally intensive data augmentation techniques. In summary, MedAlmighty illustrates the promise of leveraging knowledge distillation to combine the complementary strengths of large and small models in the medical domain. This work lays a foundation for future research into hybrid model architectures, with the goal of improving diagnostic accuracy and enabling practical, scalable deployment of AI systems in real-world healthcare settings.

## 6 Discussion

The application of large-scale vision models in medical image classification has shown significant promise, but challenges persist, particularly in scenarios with limited labeled data. Although deep learning algorithms have made substantial progress, these models typically require large volumes of annotated data for effective training. Acquiring such data in the medical field is often time-consuming and difficult, making the improvement of model performance in data-scarce environments a crucial area of research. Large-scale vision models excel at feature extraction and representation learning, enabling them to capture intricate textures and complex features in medical images. These models have demonstrated success in discriminative tasks across a variety of disease domains by leveraging hierarchical feature representations. Moreover, through pre-training on extensive general image datasets, large-scale vision models can benefit from transfer learning, thereby enhancing their feature extraction capabilities for medical image tasks.

This study investigates the use of large-scale vision models to distill knowledge into smaller models, focusing specifically on ResNet distillation. Knowledge distillation facilitates the transfer of knowledge from a larger model to a more compact one, improving efficiency and computational performance. By utilizing the strengths of large vision models, our approach enhances disease classification performance while addressing the challenges associated with deploying large models. MedAlmighty, which combines the advantages of large vision models and compact CNNs, provides a versatile solution for medical image classification, especially when dealing with limited data and complex patterns. Importantly, MedAlmighty does not rely on data augmentation techniques. Instead, it employs simple multistep learning rate adjustments. Despite this simplicity, it consistently outperforms both ResNet50 and DINOv2 individually, showcasing its substantial potential. By using a large vision model as the teacher and guiding the student model through knowledge distillation, MedAlmighty effectively transfers valuable knowledge while maintaining the parameter efficiency of the smaller model. This strategy bridges the gap between generalization capabilities and computational efficiency, presenting a highly promising approach for medical image analysis.

Overall, MedAlmighty represents an innovative solution to the challenges posed by limited data and diverse imaging patterns in medical diagnostics. Its performance on the MedMNIST v2 dataset demonstrates the effectiveness of using large vision models as teachers, enhancing classification performance and discrimination capabilities in specific disease categories. These results underscore the potential of large-scale vision models in improving medical image classification, particularly for targeted disease categories within the MedMNIST v2 dataset. While the results are promising, further research is needed to evaluate the effectiveness of this approach across diverse datasets and real-world scenarios. Extending this evaluation will help validate the approach and explore its broader applicability.

## Data Availability

Publicly available datasets were analyzed in this study. This data can be found here: (Yang et al. 2023).
